# Influence of a patient navigation program on timeliness of care in patients with esophageal cancer

**DOI:** 10.1002/cam4.5882

**Published:** 2023-04-19

**Authors:** Nikita Arora, Marcus Lo, Nader M. Hanna, Jennifer Pereira, Geneviève Digby, Robert Bechara, Shaila J. Merchant, Wilma Hopman, Shirley Li, Andrew Giles, Wiley Chung

**Affiliations:** ^1^ Division of General Surgery, Department of Surgery Queen's University Kingston Ontario Canada; ^2^ School of Medicine, Faculty of Health Sciences Queen's University Kingston Ontario Canada; ^3^ Esophagogastric Diagnostic Assessment Program Cancer Centre of Southeastern Ontario Kingston Ontario Canada; ^4^ Division of Respirology, Department of Medicine Queen's University Kingston Ontario Canada; ^5^ Division of Gastroenterology, Department of Medicine Queen's University Kingston Ontario Canada; ^6^ Department of Public Health Sciences Queen's University Kingston Ontario Canada; ^7^ Kingston Health Sciences Center Decision Support Kingston Ontario Canada; ^8^ Division of Thoracic Surgery, Department of Surgery Queen's University Kingston Ontario Canada

**Keywords:** esophageal cancer

## Abstract

**Background:**

Patient navigation (P.N.) is designed to eliminate barriers to care. The objective of this study was to evaluate the impact of a novel P.N. program on timeliness of care in patients with esophageal cancer.

**Methods:**

This retrospective study compared the timeliness of care for esophageal cancer patients before (January 2014–March 2018) and after the implementation of a novel P.N. program (April 2018–March 2020), called EDAP, at a tertiary care center. The primary outcome was time from biopsy to first treatment; secondary outcomes included time from biopsy to complete staging, biopsy to complete preoperative workup, and referral to the first point of contact. The outcomes were evaluated in the entire cohort and then in a subgroup of patients undergoing curative multimodality therapy.

**Results:**

There were 96 patients in the pre‐EDAP group and 98 patients in the post‐EDAP group. There was no significant difference between pre‐ and post‐EDAP in the time from biopsy to first treatment and time from biopsy to staging in the overall cohort. In the subgroup of patients undergoing curative multimodality therapy, there was a significant decrease in time from biopsy to first treatment postnavigation (60–51 days, *p* = 0.02), in addition to a significant decrease in time from biopsy to preoperative workup and time from biopsy to staging.

**Conclusions:**

This is the first study demonstrating that a novel P.N. program for patients with esophageal cancer improved timeliness of care. The group of patients who benefited most were those undergoing curative multimodality therapy, likely given the extensive coordination of services required by this group.

## Graphical abstract

This study demonstrates that a novel patient navigation program improved timeliness of care for patients with esophageal cancer, especially in those who are receiving multimodality treatment.

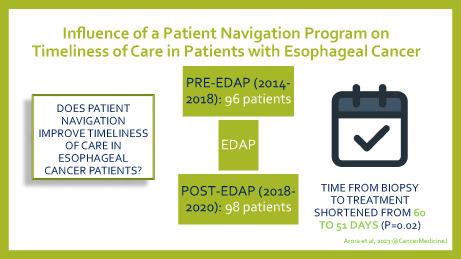

## 1 INTRODUCTION

Patient navigation (P.N.) is an intervention designed to eliminate barriers to care through the adjunctive provision and coordination of various patient‐centered services. These services can include locating low‐cost or logistically convenient screening programs, facilitating transportation to appointments, finding physicians and scheduling appointments, providing discussion and education regarding medical treatments, and overcoming personal beliefs associated with poor outcomes.^1^ The initial P.N. programs were created in response to the 1989 *Report to the Nation on Cancer in the Poor* to target the care disparities experienced by individuals of a lower socioeconomic status.^1^ However, most of the P.N. literature focuses on breast, colorectal, and cervical cancers,^1^ and there are no studies on P.N. in patients with esophageal cancer. This is likely in relation to a number of issues, including the comparatively low incidence of esophageal cancer compared to the aforementioned cancers, and difficulty with patient recruitment given the often advanced nature of esophageal cancer on presentation. Yet, patients with esophageal cancer are often disproportionately from a lower socioeconomic status,^2^ and as such, may primarily derive benefit from P.N.^3^

A recent systematic review^1^ demonstrated that P.N. significantly improves screening and diagnosis rates in certain oncology patients. However, there is little evidence as to whether P.N. can improve outcomes in the remainder of the cancer care continuum, including during staging and treatment. Some studies suggest that P.N. is associated with increased patient satisfaction^4^, ^5^, ^6^, ^7^ and higher rates of cancer therapy completion.^8^, ^9^ Meanwhile, there is mixed evidence regarding the impact of P.N. on timeliness of care. Some studies indicate that the shared decision‐making process of P.N. may prolong the time from diagnosis to first cancer treatment in prostate cancer, while others demonstrate that it shortens this time interval in pancreatic and breast cancer.^10^, ^11^, ^12^, ^13^

The objective of this study was to evaluate the impact of a novel P.N. program on timeliness of care, in patients with esophageal cancer. Specifically, we hypothesized that P.N. has the most significant impact on patients undergoing multimodality curative therapy, given that they require the greatest coordination of services during workup and treatment.

## 2 PATIENTS AND METHODS

This was a retrospective cohort study that included all patients with esophageal cancer managed at Kingston Health Sciences Centre (KHSC), a tertiary care hospital system in Kingston, Ontario. KHSC serves a predominantly rural patient population of >500,000 in southeastern Ontario. The study compares patient outcomes before and after implementing a novel P.N. model: the Esophagogastric Diagnostic Assessment Program (EDAP). We received approval through the institutional registered ethics board (#6029600, approved May 16, 2022). In addition, we ensured the patients had not withdrawn consent for research in the electronic medical record.

To identify consecutive patients with esophageal cancer managed at KHSC prior to EDAP implementation (January 2015–March 2018), we used multiple sources, including the surgeon and endoscopist's databases of all patients on whom they performed procedures during the study time period and the hospital‐based Canadian Institute for Health Information Discharge Abstract Database (DAD). The hospital‐based DAD contains all inpatient and outpatient records for patients at Kingston Health Sciences Center. The ICD‐10 code of C15 was used to identify patients from the DAD. To identify consecutive patients who received P.N. following the implementation of the EDAP (April 2018–March 2020), we used the EDAP Nurse Navigator's database in addition to the above sources. The pre‐EDAP evaluation period was longer than the post‐EDAP period, to equalize the sample sizes in the pre‐ and post‐EDAP data sets. The patients in the pre‐EDAP period who initially did not receive navigation but who were subsequently enrolled in the EDAP were excluded to clarify the treatment effect. Similarly, two patients who underwent both endoscopic treatment and esophagectomy were excluded for the same reason.

### 2.1 Description of program

The EDAP model was launched at KHSC in April 2018. All patients with esophageal cancer seen at KHSC are enrolled in this program. Patients are referred to EDAP by primary care providers and endoscopists, including family physicians, general surgeons, and gastroenterologists. Referrals for esophageal cancer that are sent to the Cancer center or the Department of Surgery are redirected to the EDAP. The EDAP referral criteria are any patient with a suspected or confirmed diagnosis of esophageal cancer or gastro‐esophageal junction (GEJ) cancer (encompasses all tumors with an epicenter within 5 cm proximal or 2 cm distal to the GE junction). Although the treatment approach to Siewert II GEJ cancers continues to be controversial, these cancers are treated as esophageal cancers at our institution.^14^ Siewert III GEJ cancers are treated as gastric cancers and not included in this study. In this model, the referral is initially triaged by a thoracic surgeon. Subsequently, a P.N. nurse contacts all patients referred to the EDAP within two weekdays from receipt of the referral. Patients were predominantly seen as outpatients; although some were seen as inpatients. The roles of the P.N. nurse include: providing informal psychosocial support during key milestones in patient care (i.e. initial consultation, endoscopy, staging, surgical consent, postoperative visits, and disclosure of recurrence), providing adjunctive education, facilitating the coordination of services and continuity of care, expediting referral pathways and communication with other health‐care providers, as well as serving as a resource for patient questions or concerns between medical appointments.

### 2.2 Study outcomes

We collected data from the electronic medical record of the KHSC health system. Included variables were patient characteristics (sex, age, and smoking/alcohol history), disease characteristics (histopathology, multifocal vs. unifocal nature of disease, Siewert classification [classification of gastro‐esophageal junction cancers], pathologic stage), treatment characteristics (treatment intent, treatment type), as well as dates of key diagnostic and treatment milestones.

**FIGURE 1:**
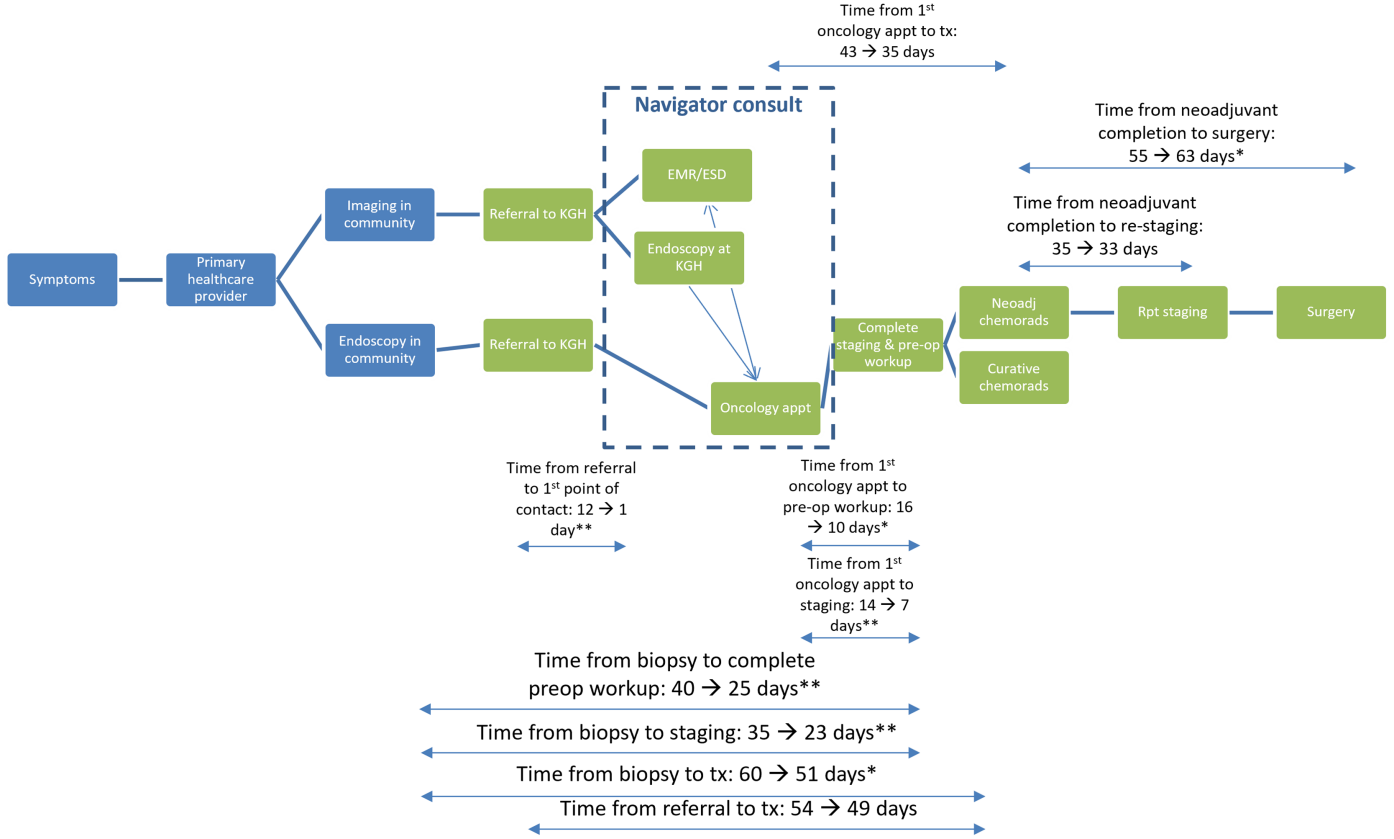
This is a visual representation of esophageal cancer patients' trajectory at our institution, as well as the changes in time intervals identified pre‐ and post‐EDAP implementation, specifically for the group under curatve multimodality therapy. Appt, appointment; Preop, preoperative; Tx, treatment. *p < 0.05, **p < 0.01.

The primary outcome was time from biopsy to any first treatment. Other outcomes were time from biopsy to complete staging, biopsy to complete preoperative workup, referral to the first point of contact, referral to complete staging, referral to any first treatment, first oncology appointment to complete staging, first oncology appointment to complete preoperative workup, first oncology appointment to any first treatment, complete neoadjuvant treatment to restaging, and complete neoadjuvant treatment to surgery (Figure 1).

The timeliness of care intervals were defined as per Cancer Care Ontario (CCO) guidelines.^15^ Complete staging was defined as completion of endoscopy, biopsy confirming malignancy, whole‐body positron emission tomography (PET), as well as computed tomography (CT) chest, abdomen, and pelvis. Preoperative workup was defined as complete staging and any additional investigations required to determine surgical candidacy. Examples of additional investigations include pulmonary function tests (PFTs), biopsies of suspicious lymph nodes, or potential metastatic lesions, echocardiograms etc. The date of referral was defined as the date on which the referral was sent by the referring physician. The date of treatment was defined as any first treatment, including endoscopic mucosal resection (EMR), endoscopic submucosal dissection (ESD), chemoradiation, or definitive surgery. Restaging after completion of neoadjuvant therapy was defined as repeat endoscopy and repeat PET or CT. The date of the first oncology appointment included an appointment with either the surgical, medical, or radiation oncologist, whichever came first. Lastly, the first point of contact refers to the first contact with the Cancer center, including with a nurse navigator, radiation oncology, medical oncology, endoscopy, or surgical oncology consult.

### 2.3 Analysis

Data were collected in an electronic spreadsheet and imported into IBM SPSS (version 27.0 for Windows, Armonk, New York, 2021) for statistical analyses. Distributions of the continuous data were assessed using the Shapiro–Wilk test. Chi‐square and Fisher's exact tests were used to compare the proportions of categorical variables between the pre‐ and post‐EDAP period. Mann–Whitney *U* tests were used to compare continuous data, which were reported using medians and interquartile range (IQR). A *p* value of <0.05 was considered statistically significant, and no adjustment was made for multiple comparisons.

We compared the study outcomes between pre‐ and post‐EDAP in the entire cohort. We then compared the study outcomes in the subgroup receiving multimodality curative therapy (excluding palliative treatment, no treatment, and EMR/ESD), pre‐ and post‐EDAP. The multimodality group was selected, as these patients require multiple investigations and oncology consultations to comply with guidelines and to ultimately undergo treatment. As such, it was felt that P.N. would likely impact this group most, by streamlining and expediting the workup and treatment for this group.

## 3 RESULTS

We identified 194 patients in total. Most patients were male (78%), the median age was 68, and adenocarcinoma was the most common histopathology (75%). There were 96 patients in the pre‐EDAP group and 98 patients in the post‐EDAP group (Table 1).

**TABLE 1:** Descriptive characteristics of entire cohort.

	Pre‐EDAP (*n* = 96)	Post‐EDAP (*n* = 98)	*p* value
Male sex—*N* (%)	73 (76)	78 (80)	0.55
Age–median (IQR)	68 (59,76)	68 (62,78)	0.32
Histopathology—*N* (%)			0.62
Adenocarcinoma	74 (77)	72 (74)	
Squamous	19 (20)	22 (22)	
Other	3 (3)	2 (2)	
Unclear	0 (0)	2 (2)	
Smoking—*N* (%)	73 (77)	59 (63)	0.04*
Alcohol—*N* (%)	57 (64)	60 (67)	0.64
Multifocal—*N* (%)	6 (6)	5 (5)	0.73
Siewert class documented—*N* (%)			<0.01**
I	19 (20)	43 (44)	
II	6 (6)	19 (19)	
Not GEJ	14 (15)	19 (19)	
Unclearly	57 (59)	17 (17)	
documented			
Treatment intent—*N* (%)			
Curative	52 (54)	57 (58)	0.19
Palliative	32 (33)	36 (37)	
No treatment	12 (13)	5 (5)	
Surgery—*N* (%)	26 (27)	26 (27)	0.93
Pathologic staging—*N* (%)			
0	13 (14)	0 (0)	<0.01**
IA	0 (0)	1 (1)	
IB	1 (1)	1 (1)	
IC	1 (1)	0 (0)	
IIA	0 (0)	4 (4)	
IIB	2 (2)	9 (9)	
IIIA	1 (1)	0 (0)	
IIIB	6 (6)	6 (6)	
IVA	3 (3)	4 (4)	
N/A	69 (72)	73 (75)	
Curative intent chemoradiation—*N* (%)	8 (15)	11 (19)	0.53
Endoscopic treatment—*N* (%)	10 (10)	16 (16)	0.23

Abbreviations: EDAP, esophageal diagnostic assessment program; IQR, interquartile range.

* *p* < 0.05

** *p* < 0.01, Chi‐square test was used to compare pre‐ and post‐navigation values, except in the cases of “histopathology,” and “pathologic staging,” where values less than five were encountered, and in these cases, Fisher's exact test was used.

The timeliness of care pre‐ and post‐EDAP for the entire cohort is summarized in Table 2. The median time from biopsy to first treatment was not significantly different between groups in the entire cohort (56 to 51 days, *p* = 0.94). The median time between biopsy to complete preoperative workup was significantly decreased post‐EDAP (41 to 25 days, *p* < 0.01). Notably, the median time from referral to the first point of contact was significantly decreased postnavigation (9 to 1 day, *p* < 0.01). Further, the median time between the first oncology appointment to complete staging (11 to 7 days, *p* = 0.01) and the median time between 1st oncology appointment to complete preoperative workup were reduced (17–10 days, *p* < 0.01). The time between neoadjuvant completion and surgery increased from 55 to 63 days, *p* = 0.02.

**TABLE 2:** Median time intervals for pre‐ and post‐EDAP for entire cohort.

	Pre‐EDAP in days (*n*)		Post‐EDAP in days (*n*)		*p* value
	Median (IQR)	*N*	Median (IQR)	*N*	
Biopsy to complete staging	28 (9,42)	75	22 (13,31)	70	0.09
Biopsy to complete preoperative workup	41 (28,49)	30	25 (18,33)	30	<0.01**
Biopsy to first treatment	56 (38,70)	77	51 (38,82)	89	0.94
Date of referral to 1st point of contact	9 (4,16)	73	1 (0,5)	83	<0.01**, *
Date of referral to complete staging	25 (3,38)	59	16 (11,27)	64	0.22
Date of referral to first treatment	52 (30,63)	59	45 (34,60)	78	0.65
Date of 1st oncology appointment to complete staging	11 (0,22)	78	7 (0,13)	71	0.01*
Date of 1st oncology appointment to complete preoperative workup	17 (11,28)	31	10 (3,17)	30	<0.01**
Date of 1st oncology appointment to first treatment	39 (21,50)	80	34 (21,50)	90	0.92
Complete neoadjuvant treatment to restaging	35 (23,42)	21	33 (28,36)	29	0.86
Complete neoadjuvant treatment to surgery	55 (51,63)	23	63 (59,74)	25	0.02*

Abbreviations: EDAP, esophageal diagnostic assessment program; IQR, interquartile range.

* *p* < 0.05

** *p* < 0.01.

We also analyzed the timeliness of care pre‐ and post‐EDAP in the subset of patients who received multimodality curative therapy (Table 3, Figure 1). The time from biopsy to the first treatment was reduced by 9 days in this subgroup (60–51 days, *p* = 0.02). Additionally, there was a significant improvement in timeliness of care seen for all of the following intervals: time from biopsy to complete staging (35–23 days, *p* < 0.01), time from biopsy to complete preoperative workup (40–25 days, *p* = 0.01), time from referral to 1st point of contact (12–1 day, *p* < 0.01), time from referral to complete staging (30–17 days, *p* < 0.01), time from 1st oncology appointment to complete staging (14–7 days, *p* < 0.01), time from 1st oncology appointment to complete preoperative workup (16–10 days, *p* = 0.01), and time from 1st oncology appointment to first treatment (43–35 days, *p* = 0.05).

**TABLE 3:** Median time intervals for pre‐ and post‐EDAP for patients receiving multimodality curative therapy (excluding EMR/ESD/palliative/no treatment).

	Pre‐EDAP in days (n)		Post‐EDAP in days (n)		*p* value
	Median (IQR)	N	Median (IQR)	N	
Biopsy to complete staging	35 (26,44)	40	23 (14,33)	39	<0.01**
Biopsy to complete preoperative workup	40 (28,47)	29	25 (18,33)	30	0.01*
Biopsy to first treatment	60 (51,70)	42	51 (40,61)	40	0.02*
Date of referral to 1st point of contact	12 (7,15)	30	1 (0,5)	39	<0.01**
Date of referral to complete staging	30 (25,42)	29	17 (13,29)	38	<0.01**
Date of referral to first treatment	54 (47,65)	30	49 (39,60)	38	0.20
Date of 1st oncology appointment to complete staging	14 (10,28)	41	7 (1,15)	40	<0.01**
Date of 1st oncology appointment to complete preoperative workup	16 (11,28)	30	10 (3,17)	30	0.01*
Date of 1st oncology appointment to first treatment	43 (34,56)	43	35 (28,47)	40	0.05
Complete neoadjuvant treatment to re‐staging	35 (23,42)	21	33 (28,36)	29	0.86
Complete neoadjuvant treatment to surgery	55 (51,63)	23	63 (59,74)	25	0.02*

Abbreviations: EDAP, esophageal diagnostic assessment program; IQR, interquartile range.

* *p* < 0.05

** *p* < 0.01.

### 3.1 Comment

Implementation of a P.N. program for patients with esophageal cancer seen at a tertiary care center improved the timeliness of care, especially in the specific subgroup of patients undergoing multimodality curative therapy. This program consisted of psychosocial support, adjunctive education, coordination of services, as well as expedited and streamlined referrals.

The primary variable of interest, time from biopsy to first treatment, significantly decreased from 60 to 51 days in the group receiving multimodality curative therapy. Notably, this study outcome was not significantly different between pre‐ and post‐EDAP in the entire cohort, affirming our hypothesis that P.N. may have the most utility for the patients undergoing curative multimodality therapy. We postulate that the mechanism by which EDAP decreases this treatment interval is by streamlining the time to completion of staging and preoperative workup, which were both significantly reduced in the postnavigation group. The investigations needed to complete preoperative workup, especially, may be varied for each patient. For example, this may include PFTs, echocardiograms, and cardiopulmonary exercise testing, which may be more likely to be missed or delayed without the assistance of a navigator. Interestingly, we found that time to surgery after neoadjuvant completion was increased postnavigation (from 55 to 63 days). It is not clear why this happened, but it may be due to increased patient volumes and decreased surgical resources over time. Nonetheless, the time between neoadjuvant completion and surgery remains within the acceptable time interval to perform surgery after neoadjuvant therapy, as per treatment guidelines.^16^ In fact, the rates of complete pathologic response to neoadjuvant treatment are higher in those who wait longer after neoadjuvant completion to undergo surgery, up to 64–98 days.^17^, ^18^

Our results are similar to those of Habbous et al,^19^ which demonstrated a median time from biopsy to first treatment of 46 days, compared to 51 days in the post‐navigation cohort at our institution. However, in contrast to our study, Habbous et al^19^ also included patients undergoing palliative treatment, which may have led to the observed differences in times. Specifically, the shortest biopsy to first treatment interval is often for those receiving palliative radiation, and as such, this may have shortened their average biopsy to first treatment interval. Additionally, the Habbous et al^19^ study notes that the time of diagnosis used in their study may have been inaccurate for certain patients in the cohort. Our study validated the time of diagnosis by specifically reviewing endoscopy and pathology reports for all included patients. In the broader context, wait time data worldwide is significantly varied. A study from the Netherlands demonstrated a 42.7–48.3 day wait time from diagnosis to treatment,^20^ whereas, at select expert centers, United States data demonstrates even shorter wait times of 16–27 days in one study^21^ and 30–37 days in another.^22^ Each of these contexts is significantly different from our center, in terms of resource availability, infrastructure, and geographical spread of patients, making it difficult to make direct comparisons.

When seeking to compare our study results to broader benchmarks, we found an absence of national or provincial benchmarks for timeliness of care specifically for patients with esophageal cancer, given the significantly varied health‐care infrastructure in differing jurisdictions. For example, in Ontario, the benchmark set out by Health Quality Ontario for the time between referral and surgeon consultation for any cancer is 10 days. P.N. allowed us to exceed this provincial benchmark by decreasing the time from referral to the first point of contact from 12 days to 1 day. Although the first point of contact was not always a surgeon, this shortened waiting period likely provides psychosocial benefit to patients, as demonstrated in studies evaluating navigation in other disease sites.^23^ Further, the national benchmark for wait times for radiation therapy is four weeks.^24^ P.N. allowed for a decrease in wait time for neoadjuvant chemotherapy/radiation from 6 to 5 weeks (time from first oncology appointment to first treatment). As such, the program allowed us to move closer to national benchmarks for radiation treatment, although definitions of time intervals differ slightly between guidelines and this study's definitions.

Although it is notable that the EDAP reduced various diagnostic and treatment time intervals, there is varied evidence regarding the significance of wait times on the care trajectory of patients with esophageal cancer. Several studies have specifically investigated the effect of time from symptom onset to diagnosis^25^, ^26^ and diagnosis to treatment^20^, ^27^ and found no impact on overall survival or disease‐free survival. Yet, Grotenhuis et al^26^ demonstrated that shortening the time from diagnosis to treatment decreases in‐hospital mortality and morbidity. Despite the evidence being equivocal regarding the broader impact of timeliness on prognosis and survival in esophageal cancer, longer wait times have been demonstrated to poorly affect mental health, especially in patients with pre‐existing psychiatric disorders.^28^ Moreover, timeliness has been shown to be an indicator of the quality of care in a health‐care system in general.^27^

The next steps for this body of work include evaluating the impact of the EDAP P.N. program on other domains of health quality, including the impact on patient quality of life, emergency department use, and guideline compliance.

## 4 LIMITATIONS

There are several limitations to this study. First, this study was a pre‐post comparison study; as such, there may have been other factors unaccounted for that influenced timeliness of care over the study period. There may have been significant changes in surgical resource allocation, referral volumes, and oncologist/surgeon practices over time, which we were not able to assess or control for in this study. Moreover, there were some differences in the pre‐ and post‐navigation groups identified in our study in Table 2, such as significant differences in Siewert class, pathologic staging, and smokers. However, these differences are mechanistically unlikely to have affected the study findings and likely reflect changes in disease profiles and documentation over time. It was not feasible to adjust for these differences using propensity scoring given the small sample sizes available. Second, we do not perform an endoscopic ultrasound as part of routine staging at our institution, thereby limiting our ability to collect specific clinical T‐ and N‐staging information and control for this variable in our analysis. At the same time, this may have expedited our overall time to first treatment by eliminating a component of the required staging workup. Third, we were able to access some information regarding diagnostic tests and oncology appointments which our patients attended at outside institutions; however, we could not access these outside institutions' complete electronic medical records and, therefore, may be missing some information. Fourth, our institution's results may have limited applicability to the results of other institutions' P.N. programs, as the components of a P.N. program may vary significantly between institutions.

## 5 CONCLUSION

This is the first study demonstrating that a novel P.N. program for patients with esophageal cancer improved the timeliness of care. Further, it is one of few studies that assess a P.N. program involved in the diagnosis and treatment components of the patient's oncologic journey, as most previous studies focus on improving screening and diagnosis. This study validated the accuracy of the data by confirming the dates via chart review, in contrast to other large population studies,^19^ in which accuracy is less certain. Lastly, we were able to distinguish the subgroup of patients who are most likely to benefit from P.N.: those who receive multimodality curative therapy, as opposed to those who undergo EMR/ESD and/or palliative treatment. Further work must be done to elucidate the holistic impact of this program on the treatment journeys of patients with esophageal cancer, the subsets of patients that benefit the most from this program (such as patients of a lower socioeconomic status), and how this program can be generalized across jurisdictions.

## AUTHOR CONTRIBUTIONS

**Nikita Arora:** Conceptualization (lead); data curation (lead); formal analysis (lead); investigation (lead); methodology (lead); project administration (lead); visualization (lead); writing – original draft (lead); writing – review and editing (lead). **Marcus Lo:** Data curation (equal); writing – review and editing (equal). **Nader M. Hanna:** Investigation (equal); writing – review and editing (equal). **Jennifer Pereira:** Conceptualization (equal); project administration (lead); resources (equal); writing – review and editing (equal). **Genevieve Digby:** Writing – review and editing (equal). **Robert Bechara:** Data curation (equal); writing – review and editing (equal). **Shaila J. Merchant:** Data curation (equal); writing – review and editing (equal). **Wilma Hopman:** Formal analysis (equal); validation (equal); writing – review and editing (equal). **Shirley Li:** Data curation (equal); writing – review and editing (equal). **Andrew Giles:** Writing – review and editing (equal). **Wiley Chung:** Conceptualization (equal); data curation (equal); methodology (equal); resources (equal); supervision (lead); writing – review and editing (equal).

## FUNDING INFORMATION

Division of Thoracic Surgery, Queen's University.

## CONFLICT OF INTEREST STATEMENT

None.

## ETHICS STATEMENT

Registered ethics board approval was attained from our center.

## PATIENT CONSENT

This was not directly required, but no patients who had withdrawn consent from research were included in the study.

## Data Availability

The data that support the findings of this study are available on request from the corresponding author. The data are not publicly available due to privacy or ethical restrictions.
